# Isolation and characterization of twelve polymorphic microsatellite markers in the endangered *Hopea hainanensis* (Dipterocarpaceae)

**DOI:** 10.1002/ece3.7077

**Published:** 2020-12-02

**Authors:** Chen Wang, Xiang Ma, Liang Tang

**Affiliations:** ^1^ Key Laboratory of Tropical Biological Resources of Ministry of Education School of Life and Pharmaceutical Sciences Hainan University Haikou China; ^2^ College of Ecology and Environment Hainan University Haikou China

**Keywords:** Dipterocarpaceae, endangered species, *H. hainanensis*, microsatellite markers, next‐generation sequencing

## Abstract

Microsatellite markers were isolated and characterized for *Hopea hainanensis* Merrill & Chun, an endangered tree species with scattered distribution in Hainan Island and northern Vietnam. Twenty‐six microsatellite markers were developed based on next‐generation sequencing data and were genotyped by capillary electrophoresis on an ABI 3730xl DNA Analyzer. Twelve markers were found to be polymorphic in *H. hainanensis*. GENODIVE analyses indicated that the number of alleles ranged from 2 to 6 per locus, and the observed and expected heterozygosity varied from 0 to 0.755 and from 0.259 to 0.779, respectively. Primer transferability was tested with *Hopea chinensis* Hand.‐Mazz. and *Hopea* *reticulata* Tardieu, in which 3 and 7 microsatellite markers were found to be polymorphic, separately. The results showed that *H. reticulata* and *H. hainanensis* had similar levels of genetic diversity. A neighbor joining dendrogram clustered all individuals into two major groups, one of which was exclusively constituted by *H. hainanensis*, while the other consisted of two subgroups, corresponding to *H. reticulata* and *H. chinensis*, respectively. The 12 polymorphic microsatellite markers could be applied to study genetic diversity, population differentiation, mating system, and fine‐scale spatial genetic structures of *H. hainanensis* as well as its close relatives, facilitating the conservation and restoration of these endangered but valuable *Hopea* species.

## Graphical abstract

We reported, for the first time, twelve polymorphic microsatellite markers for an endangered tree species *Hopea hainanensis* Merrill & Chun. These newly developed microsatellite markers could be applied to study genetic diversity, population structure, and mating system of *H. hainanensis*, as well as its close relatives, facilitating the conservation and restoration of these endangered and valuable *Hopea* species.

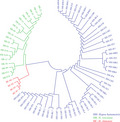

## 1 INTRODUCTION

*Hopea hainanensis* Merrill & Chun is a large evergreen tree that can grow up to 20 m. It is found in tropical lowland forest of Hainan Island and northern Vietnam (Li et al., 2007). *Hopea hainanensis* is known for its highly valued timber which is extremely durable and suitable for making boats and building bridges and houses (Li et al., 2007). As a result, adult trees of this species had been overly logged, leading to a reduction of 50%–70% population in the last three hundred years (Ly et al., 2018). The remaining population of *H. hainanensis* is severely fragmented and isolated in a few reserves in Hainan Island. This species is scarce in its natural habitat and is assessed as endangered according to the IUCN Red List of Threatened Species (Ly et al., 2018). In addition to the highly valued wood, *H. hainanensis* is rich in bioactive compounds. The extracts from stems and barks were reported to have potent antioxidant activities, which could be used as candidates for pharmaceutical products or food additives (Ge et al., 2009).

*Hopea hainanensis* belongs to the family Dipterocarpaceae, which comprises 16 genera and more than 500 species (Ashton, 1988). Trees of this family dominate Southeast Asia's tropical forests, accounting for 20%–50% of forest basal area and often well over 50% of canopy trees (Ashton, 1988; Ghazoul, 2016). Many species of this family constitute important timber resources and thus have been heavily exploited by local countries in tropical Asia. The unsustainable exploitation for timber and deforestation for agriculture render many dipterocarp species now being classified as endangered (Ghazoul, 2016). Understanding the genetic diversity, population structure and mating system of these endangered species is crucial and of priority for the effective management and conservation (Frankham, 1995). Population genetic studies focused on dipterocarp species have been carried out for the purpose of conservation and restoration (Finger et al., 2012; Ismail et al., 2014). Microsatellite markers are widely used to estimate genetic diversity, fine‐scale spatial genetic structure, gene flow, and mating system for endangered species in Dipterocarpaceae (Finger et al., 2012; Lee et al., 2013; de Morais et al., 2015). However, the development of informative microsatellite markers is first step in population genetic studies. Indeed, microsatellite loci have been isolated and characterized for species in genera *Shorea*, *Vatica*, *Dipterocarpus*, *Neobalanocarpus*, and *Dryobalanops* (Guo et al., 2017; Isagi et al., 2002; Iwata et al., 2000; Lee et al., 2004b; Nanami et al., 2007). Lee et al. (2004a) developed SSR markers for *Hopea bilitonensis* from dinucleotide repeats‐enriched genomic library and validated 15 of them across 24 adult trees, and however, they did not investigate the transferability of these SSR primers.

In this study, we sequenced the genome of *H. hainanensis* using next‐generation sequencing technology. Based on the assembled contigs, 26 novel microsatellite markers were developed and characterized using 50 individuals of this species, 12 of which were found to be polymorphic. The marker transferability was tested upon two additional *Hopea* species, *H. chinensis* Hand.‐Mazz. and *H. reticulata* Tardieu. These newly developed microsatellite markers could be used as a universal tool in population genetic studies of *H. hainanensis* as well as its close relatives.

## 2 MATERIALS AND METHODS

Fifty individuals of *H. hainanensis* were collected from 10 natural populations at Hainan Island, China for primer testing and diversity assessment (Table 1). Two additional species of genus *Hopea*, *H. chinensis* and *H. reticulata*, were included for cross‐species amplification (Table 1). Voucher specimens of the studied species were deposited in Hainan University, Haikou, China (Herbarium code: HUTB). Whole genomic DNA was extracted from silica gel‐dried leaf tissues using the DNeasy Plant Mini Kit (QIAGEN, Shanghai, China). The genomic DNA of one *H. hainanensis* sample (Voucher code: Tang161207) collected from Jianfeng Mountain in Hainan Island was used for Illumina Paired‐end sequencing. A genomic DNA library with 350–450 bp inserts was constructed with a TruePrep DNA Library Prep Kit V2 and then was sequenced by an Illumina HiSeq 2500 system using the 2 × 250‐bp read mode at JINTAI Biotech. Raw sequencing data were filtered with Trimmomatic to remove adaptor sequences and low‐quality bases with default parameters (Bolger et al., 2014). Clean reads were extended and merged by overlapping paired‐end reads using FLASH with minimum and maximum overlaps of 20 and 100 bp, respectively (Magoc & Salzberg, 2011). The extended reads were clustered by CD‐HIT with the minimum identity of 98% (Fu et al., 2012). Microsatellite motifs were screened by MISA (Thiel et al., 2003) with search parameters set as follows: at least six repeats for dinucleotide motifs, five repeats for tri‐ and tetranucleotide motifs, and four repeats for penta‐ and hexanucleotide motifs. Two adjacent microsatellite motifs with base pairs less than 100 between each other were recognized as a compound microsatellite and discarded. Microsatellites with sufficiently long flanking regions were retained, based on which primers were designed and examined using Primer Premier 5.0 (Clarke & Gorley, 2001).

**TABLE 1:** Geographic origin, sample size, and voucher information for *Hopea hainanensis*, *Hopea* *reticulata*, and *Hopea chinensis* used in this study

Species	Collection locality	*n*	Geographic coordinates	Voucher No.
*Hopea hainanensis* Merrill & Chun	Limu Mountain, Hainan Province, China	5	19.1909°N, 109.7417°E	Tang171022
	Jiaxi Country, Hainan Province, China	5	18.8429°N, 109.1662°E	Tang170602
	Kafa Mountain, Hainan Province, China	5	18.6988°N, 109.3303°E	Tang180505
	Jianfeng Mountain, Hainan Province, China	5	18.7422°N, 108.9902°E	Tang161207
	Fanjia Country, Hainan Province, China	5	19.2722°N, 109.6150°E	Tang171220
	Diaoluo Mountain, Hainan Province, China	5	18.6961°N, 109.8839°E	Tang171202
	Qinwang Mountain, Hainan Province, China	5	18.9388°N, 109.4468°E	Tang170604
	Maorui Forestry Station, Hainan Province, China	5	18.6724°N, 109.4116°E	Tang180515
	Bawang Mountain, Hainan Province, China	5	19.0982°N, 109.1313°E	Tang170407
	Baolong Forestry Station, Hainan Province, China	5	18.4855°N, 109.4385°E	Tang180511
*H*.*reticulata* Tardieu	Ganshen Mountain, Hainan Province, China	20	18.3913°N, 109.6678°E	Cai191220
*H*.*chinensis* Hand.‐Mazz.	Xishuangbanna Tropical Botanical Garden, Yunnan Province, China	4	21.9272°N, 101.2559°E	Cai190712

Note

*n*: number of samples.

Voucher specimens were deposited in the Herbarium of Hainan University, Haikou, China (HUTB).

Firstly, we tested the specificity of the primers using 10 individuals of *H. hainanensis* to screen those that could generate a single clear band with the expected size. PCR amplification was carried out with an Eppendorf Mastercycler ep gradient S thermocycler (Eppendorf) in a 20 µl final reaction volume containing 1 µl gDNA (at least 50 µg/ml), 0.2 µl of each primer (50 µM), and 10 µl 2 × Taq PCR MasterMix (TIANGEN Biotech). The following cycling program was used: 5 min of denaturation at 94°C; followed by 32 cycles of denaturing at 94°C for 20 s, annealing at 50–60°C for 20 s, and extension at 72°C for 60 s, with a final extension of 7 min at 72°C. PCR products were separated in a 1.2% agarose gel to validate whether only one band with the expected size was amplified. Primer pairs with good specificity were selected and labeled with the fluorescent dye FAM, HEX, or TAMRA in the forward primers. Amplifications were performed with the fluorescent‐labeled primers under the same condition for all samples of the three *Hopea* species. The PCR products were separately combined with a GeneScan 500 LIZ Size Standard (Life Technologies) and resolved by capillary electrophoresis on an ABI 3730xl DNA Analyzer (Applied Biosystems) at the TIANYI Biotechnology Company. Capillary electrophoresis is the preferred method for SSR genotyping because of its high resolving power and good repeatability (Mason, 2015). Sizes of SSR alleles (in base pairs) were determined with GeneMarker version 2.2 (SoftGenetics) and manually corrected. To ensure the repeatability of genotyping analysis, alleles scored in only one individual were amplified and genotyped once more via independent PCR runs and capillary electrophoresis assay.

In view of the autopolyploidy nature of *H. hainanensis* and *H. reticulata* (personal communication with Rong Wang, East China Normal University, who initiated the whole genome sequencing of the two *Hopea* species), allelic dosage was analyzed based on the ratios between peak intensities following the MAC‐PR method (Esselink et al., 2004). GENODIVE version 3 was adopted to estimate genetic diversity and test deviation from Hardy–Weinberg equilibrium (HWE), as this software can take account of missing dosage information for partial heterozygotes of autopolyploid (Meirmans, 2020). Another challenge posed by autopolyploidy is polysomic inheritance, under which double‐reduction may occur and bias the results of standard population genetic analyses (Huang et al., 2019). However, genotypic ambiguities caused by unknown allelic dosage in autopolyploid could not be fully resolved with the MAC‐PR method (Esselink et al., 2004). Huang et al. (2020) developed a new software package named POLYGENE for estimating population genetic statistics directly from allelic phenotypes (electrophoresis band types). For a microsatellite locus, POLYGENE could infer the possible genotypes and their posterior probabilities based on the allelic phenotype, and then, it estimates the allele frequencies through an iterative algorithm designed by Kalinowski and Taper (2006). Therefore, population genetic analyses were further performed using POLYGENE which take into account both double‐reduction and genotypic ambiguities faced by microsatellite studies on polyploids (Huang et al., 2020). *Hopea chinensis* is a diploid species; thus, it was analyzed under the diploid model with GENODIVE and POLYGENE (Trang & Triest, 2016). Analysis of molecular variance (AMOVA, Excoffier et al., 1992) implemented in POLYGENE was performed to hierarchically partition genetic variation among *H. hainanensis* populations. A neighbor joining tree based on the chord genetic distance (Cavalli‐Sforza & Edwards, 1967) was constructed with MEGA 5.0 (Tamura et al., 2011) using all individuals of the three *Hopea* species.

## 3 RESULTS AND DISCUSSION

A total of 14,616,880 raw reads were produced by Illumina paired‐end sequencing, and 14,575,674 clean reads were obtained after trimming. The filtered sequencing data have been deposited in National Center for Biotechnology Information (NCBI) Sequence Read Archive (SRA) under accession number SRX8159711. The clean reads were merged into 6,378,098 extended reads, from which 4,453,650 clusters were generated to further remove redundancy in the sequencing data. In total, 240,929 microsatellite loci were detected, and PCR primers were successfully designed for 8,003 loci with perfect motifs, of which 4,313 were dinucleotide, 1,905 were trinucleotide, 438 were tetranucleotide, 755 were pentanucleotide, and 191 were hexanucleotide.

Eighty‐eight primer pairs were synthesized and tested by PCR amplification using 10 individuals of *H. hainanensis*. Thirty‐five primer pairs that can generate a single clear band with the expected length were labeled with the fluorescent dye FAM, HEX, or TAMRA in the forward primers. Among the 35 microsatellite loci amplified by the fluorescent‐labeled primers, 26 could be scored, of which 12 were found to be polymorphic and 14 were monomorphic. DNA sequences of the polymorphic microsatellites have been submitted to NCBI with accession numbers from MT386567 to MT386578. The genetic diversity was estimated by GENODIVE (Table 2). The number of alleles ranged from 2 to 6 with an average of 3.75 alleles per locus, while the effective number of alleles ranged from 1.157 to 2.708 with an average of 1.775 alleles per locus. The observed and expected heterozygosities ranged from 0 to 0.755 and from 0.259 to 0.779, respectively. Comparable results were obtained through POLYGENE analyses (Table 2). The observed and expected heterozygosities ranged from 0 to 0.755 and from 0.255 to 0.757, respectively. The polymorphism information content (PIC) of the 12 loci ranged from 0.222 to 0.719. Deviation from HWE was detected in a large number of loci, and the estimated inbreeding coefficients (*F*_IS_) were apparently different from zero, indicating a nonrandom mating in natural populations of *H. hainanensis*. The census population size of this species is extremely small (Ly et al., 2018). Small populations are expected to experience severe inbreeding and genetic drift, resulting in departure of HWE. Another possible contribution to departure from HWE is double reduction, which could take place during meiosis in autopolyploid (Huang et al., 2019). The negative value of *F*_IS_ observed at a few loci (HHA01 and HHA11) suggested an excess of heterozygotes, which might be caused by the stochastic nature of mutation across SSR loci (Putman & Carbone, 2014). An analysis of molecular variance (AMOVA) for *H. hainanensis* revealed that 80.0% of total genetic variation was partitioned within populations (Table 3). High proportion of variation was generally found to be maintained within populations of dipterocarp species, which is mainly attributed to outcrossing and woody nature of these species (Ghazoul, 2016).

**TABLE 2:** Characteristics and genetic diversity of 12 polymorphic microsatellite markers for *Hopea hainanensis*

Locus	Primer sequence (5′–3′)	Repeat	Size range (bp)	GENODIVE				POLYGENE					Fluorescent dye	GenBank accession no.
				*n*_a_	*n_e_*	*H*_o_ ^a^	*H*_E_	*H*_o_ ^a^	*H*_E_	PIC	I	*F*_IS_		
HHA01	F: AGTTGGAGATTAAAGAAAGTGGCT	(TTTTA)_30_	103–108	2	1.485	0.430^***^	0.339	0.430^***^	0.338	0.281	0.521	−0.274	FAM	MT386567
	R: TTCAATTTAGACCCGTGGACCTC													
HHA03	F: ACATGGTCTTTGTTATCTGCTTA	(TTCT)_28_	155–163	3	1.908	0.557^***^	0.580	0.557^***^	0.564	0.500	0.953	0.013	TAMRA	MT386568
	R: CCATGGTGCTACAACCTTTCTTG													
HHA04	F: TTCATGGTCATTGAGTCATAGGT	(AT)_20_	124–134	4	1.852	0.387^***^	0.580	0.384^***^	0.551	0.498	0.992	0.303	FAM	MT386569
	R: GCCTCTACCTAGTGTATGAAGGC													
HHA11	F: ACCTGGTAAGCCATAACACTGAA	(TTC)_18_	144–150	3	2.708	0.755^***^	0.663	0.755^***^	0.656	0.582	1.083	−0.150	HEX	MT386570
	R: TGATGCAAGCTCCAGAAACAAAG													
HHA14	F: AGTCAATGAGAAGGAGACATGTT	(TA)_16_	116–132	3	1.323	0.000^***^	0.434	0.000^na^	0.403	0.363	0.721	1.000	TAMRA	MT386571
	R: AAGTCATTTGGTAAAAGGTGCCC													
HHA24	F: GCTTTCTGCATTTCCTTGAGAGA	(AT)_18_	141–153	4	1.157	0.077^***^	0.482	0.077^***^	0.447	0.410	0.840	0.828	FAM	MT386572
	R: TGATTAGCTGCTGAATTTGGCTG													
HHA27	F: ACGAATGGAGGTTTGTAATTGGA	(AT)_20_	127–137	6	2.105	0.435^***^	0.779	0.437^na^	0.757	0.719	1.543	0.423	FAM	MT386573
	R: AGAGTACAATCGGGATCAATGGA													
HHA32	F: TCAAACGCAACATGGAATAAGGA	(AT)_18_	222–230	6	2.233	0.490^***^	0.750	0.490^***^	0.724	0.677	1.408	0.323	TAMRA	MT386574
	R: AGCCATTAACTCAGAACACGAGA													
HHA41	F: GATGAGGGATAATGGTGCGTTTG	(AAG)_15_	126–129	2	1.377	0.087^***^	0.477	0.084^***^	0.455	0.351	0.647	0.815	FAM	MT386575
	R: CAACTCACGCCTCTGTGTTATTG													
HHA49	F: TCAATCGTTTTGAACCACAGGTG	(AT)_16_	157–159	2	1.276	0.227^ns^	0.259	0.227^ns^	0.255	0.222	0.423	0.111	HEX	MT386576
	R: AGCTATTGCCTAGAAGATTTCACAC													
HHA50	F: GGCATCGTAATACCGCATAGAGA	(AT)_20_	159–165	5	1.911	0.452^ns^	0.656	0.450^***^	0.638	0.583	1.213	0.294	HEX	MT386577
	R: CTACCAACAACACTAGGCGCTGT													
HHA62	F: ATTACTAACCTTTGCCCACTCCT	(GT)_20_	78–94	5	1.969	0.560^ns^	0.598	0.554^***^	0.599	0.544	1.139	0.074	HEX	MT386578
	R: ACCAGCTTTAGCCAATTCAAACC													

Note

*n*_a_: number of alleles, *n*_e_: effective number of alleles, *H*_o_: observed heterozygosity, *H*_e_: expected heterozygosity, PIC: polymorphic information content, I: Shannon's information index, *F*_IS_: inbreeding coefficient.

^a^ Significant deviation from Hardy–Weinberg equilibrium: ^*^ *p* < .05, ^**^ *p* < .01, and ^***^ *p* < .005; ns = not significant; na = not applicable.

**TABLE 3:** Analysis of molecular variance (AMOVA) for *Hopea hainanensis* populations

Source	*df*	Sum of squares	Variance	Percentage of variation
Among populations	108	5,672.03	1.90	20.00
Among individuals within population	472	7,128.67	2.51	26.43
Within individuals	1,776	9,018.95	5.08	53.56

**FIGURE 1:**
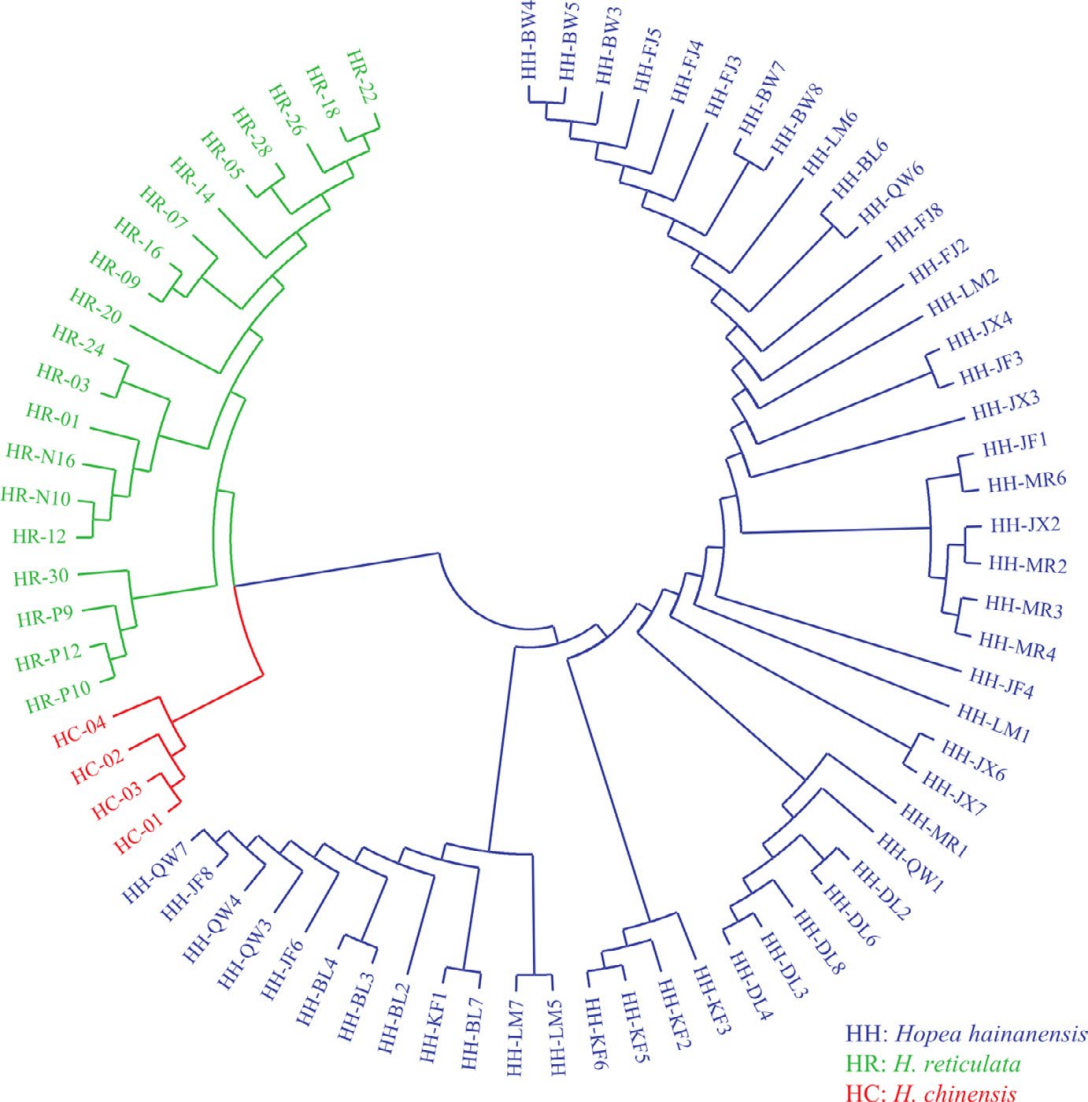
The neighbor joining tree based on the chord genetic distance constructed for all individuals of the three Hopea species.

Primer transferability was tested by cross‐species amplification in 20 and four individuals of *H. reticulata* and *H. chinensis*, respectively (Table 4). Results showed that nine SSR loci could be amplified in *H. reticulata*, among which seven were polymorphic, whereas 10 loci could be amplified in *H. chinensis*, among which three were polymorphic. *H. chinensis* was not considered in diversity comparison given such a few individuals used for primer testing. For *H. reticulata*, diversity parameters estimated by GENODIVE were close to those calculated using POLYGENE, and thus, only the results of GENODIVE were discussed (Table 4). The number of alleles ranged from 2 to 8 with an average of 3.43 alleles per locus, while the effective number of alleles ranged from 1.544 to 3.302 with an average of 2.241 alleles per locus. The observed and expected heterozygosities varied from 0 to 0.692 and 0.357 to 0.713, respectively. The polymorphism information content (PIC) ranged from 0.280 to 0.756. Deviation from HWE was detected in four loci or only in HHA03, depending on the testing methods used. Three loci (HHA04, HHA24, and HHA62) had high *F*_IS_ values, indicating an excess of homozygotes at these loci. Based on the polymorphic microsatellite markers, *H. reticulata* showed a similar level of genetic diversity compared with *H. hainanensis*. A neighbor joining dendrogram clustered all individuals into two major groups (Figure 1). One group was entirely constituted by *H. hainanensis*, while *H. reticulata* and *H. chinensis* formed two reciprocally monophyletic clades of the second group. This result suggested that the newly developed microsatellite markers could be potentially applied to differentiate species in genus *Hopea*.

**TABLE 4:** Characteristics and genetic diversity of polymorphic microsatellite markers for *Hopea reticulata* and *Hopea chinensis*

Species	Locus	Size range (bp)	GENODIVE				POLYGENE				
			*n*_a_	*n_e_*	*H*_o_ ^a^	*H*_E_	*H*_o_ ^a^	*H*_E_	PIC	*I*	*F*_IS_
*H. reticulata* (*n* = 20)	HHA01	93–98	2	1.544	0.354^ns^	0.357	0.349^ns^	0.336	0.280	0.519	−0.037
	HHA03	159–167	4	2.483	0.692^***^	0.606	0.685^***^	0.640	0.570	1.104	−0.070
	HHA04	150–154	3	2.740	0.133^***^	0.650	0.133^na^	0.635	0.559	1.049	0.790
	HHA24	143–159	8	3.302	0.282^***^	0.713	0.279^na^	0.784	0.756	1.734	0.645
	HHA27	123–125	2	1.976	0.529^ns^	0.500	0.528^na^	0.488	0.369	0.682	−0.082
	HHA41	129–135	3	2.041	0.537^ns^	0.518	0.532^na^	0.563	0.476	0.917	0.056
	HHA62	68–74	2	1.600	0.000^***^	0.387	0.000^na^	0.375	0.305	0.562	1.000
*H. chinensis* (*n* = 4)	HHA03	155–175	3	2.133	0.500^ns^	0.625	0.500^ns^	0.531	0.468	0.900	0.059
	HHA14	122–132	2	1.600	0.000^ns^	0.500	0.000^*^	0.375	0.305	0.562	1.000
	HHA27	113–123	3	2.133	0.250^ns^	0.667	0.250^ns^	0.531	0.468	0.900	0.529

Note

*n*_a_: number of alleles, *n*_e_: effective number of alleles, *H*_o_: observed heterozygosity, *H*_e_: expected heterozygosity, PIC: polymorphic information content, I: Shannon's information index, *F*_IS_: inbreeding coefficient.

^a^ Significant deviation from Hardy–Weinberg equilibrium: ^*^ *p* < .05, ^**^ *p* < .01, and ^***^ *p* < .005; ns = not significant; na = not applicable.

In conclusion, twelve novel and polymorphic microsatellite markers have been developed for the endangered species *H. hainanensis*. These co‐dominant markers can be applied to assess the genetic diversity, population structure and mating system of *H. hainanensis*, which lays foundation for efficient conservation and management of this endangered species. In addition, the successful cross‐amplification of seven and three polymorphic microsatellite markers in *H. reticulata* and *H. chinensis*, respectively demonstrates the potential application of these markers in population genetic researches of other *Hopea* species.

## CONFLICT OF INTEREST

The authors declare no conflict of interest.

## AUTHOR CONTRIBUTIONS

**Chen Wang:** Data curation (equal); investigation (lead); validation (equal). **Xiang Ma:** Funding acquisition (equal); validation (equal); writing – review and editing (equal). **Liang Tang:** Conceptualization (lead); funding acquisition (equal); methodology (lead); project administration (lead); supervision (lead); writing – original draft (lead); writing – review and editing (equal).

## Data Availability

Genomic sequences of *H. hainanensis*, NCBI SRA: SRX8159711. DNA sequences of microsatellites, GenBank accessions MT386567–MT386578. Sampling locations and microsatellite genotypes of this study are available from the Dryad Digital Repository (https://doi.org/10.5061/dryad.0gb5mkkzs).
